# The novel transcriptional regulator SA1804 Is involved in mediating the invasion and cytotoxicity of Staphylococcus aureus

**DOI:** 10.3389/fmicb.2015.00174

**Published:** 2015-03-09

**Authors:** Junshu Yang, Xudong Liang, Yinduo Ji

**Affiliations:** ^1^Department of Veterinary and Biomedical Sciences, College of Veterinary Medicine, University of MinnesotaSaint Paul, MN, USA

**Keywords:** *S. aureus*, transcriptional regulator SA1804, two-component regulatory system SaeRS, bacterial invasion, cytotoxicity

## Abstract

The two-component regulatory system, SaeRS, controls expression of important virulence factors, including toxins and invasins, which contribute to the pathogenicity of *Staphylococcus aureus*. Previously, we conducted a transcriptomics study for identification of SaeRS regulon and found that inactivation of SaeRS dramatically enhances the transcription of a novel transcriptional regulator (SA1804). This led us to question whether SA1804 is involved in bacterial pathogenicity by regulating the expression of virulence factors. To address this question, we created *sa1804, saeRS, and sa1804/saeRS double* deletion mutants in a USA300 community-acquired MRSA strain, 923, and determined their impact on the pathogenicity. The deletion of *sa1804* dramatically increased the cytotoxicity and enhanced the capacity of bacteria to invade into the epithelial cells (A549), whereas the deletion of *saeRS* eliminated the cytotoxicity and abolished the bacterial ability to invade into the epithelial cells. Moreover, the double deletions of *sa1804* and *saeRS* appeared a similar phenotype with the *saeRS* null mutation. Furthermore, we determined the regulatory mechanism of SA1804 using qPCR and gel-shift approaches. Our data indicate that the novel virulence repressor SA1804 is dependent on the regulation of SaeRS. This study sheds light on the regulatory mechanism of virulence factors and allows for us further elucidate the molecular pathogenesis of *S. aureus*.

## Introduction

*Staphylococcus aureus*, especially methicillin-resistant *S. aureus* (MRSA), is a major community and hospital acquired pathogen that can cause superficial infections, including skin and soft tissue lesions (Lowy, 1998), as well as life-threaten infections, such as pneumonia, endocarditis, and toxic shock syndrome (Klevens et al., 2007; Gordon and Lowy, 2008). The ability of this organism to cause such a variety of diseases partially depends on the expression of virulence factors that allow the bacteria to colonize and evade the host immune system.

*S. aureus* produces various surface-associated proteins to facilitate bacterial colonization and invasion (Foster and Höök, 1998). Fibronectin-binding proteins are the main surface-associated proteins of bacterial cells that function as invasins by assembling the extracellular matrix protein Fn that bridges to the host cell receptors such as α_5_β_1_-integrin, which in turn lead to bacterial internalization of host cells (Sinha et al., 1999; Fowler et al., 2000). On the other hand, the exported α-toxin is an important virulence factor of *S. aureus*. α-toxin can cause cell apoptosis and death via different signaling transduction pathways (Essmann et al., 2003; Liang and Ji, 2007).

Two-component signal transduction regulatory systems (TCSs) have been implicated together with other regulators to mediate the pathogenicity of *S. aureus* (Novick et al., 1993; Giraudo et al., 1999; Montgomery et al., 2010). The expression of virulence factors is coordinately controlled by TCSs, such as Agr (Novick, 2003), ArlRS (Fournier et al., 2001; Liang et al., 2005), and SaeRS (Giraudo et al., 1999; Liang et al., 2006), and transcriptional regulators, such as SarA (Cheung et al., 1992), Rot (McNamara et al., 2000), and Mgr (Luong et al., 2003). The SaeRS system plays a crucial role in the control of virulence gene expression (Liang et al., 2006; Rogasch et al., 2006) and in biofilm formation (Mrak et al., 2012; Olson et al., 2013). SaeRS not only up-regulates the transcription of *hla*, *hlb,* and *coa* (Giraudo et al., 1999)*,* and nuclease (*nuc*) production *in vitro* (Olson et al., 2013), but also controls *hla* expression *in vivo*, as the transcriptional level of *hla* remarkably decreased during infection with *sae* mutants (Goerke et al., 2001, 2005). Moreover, the SaeRS-dependent and Agr/SarA-independent activation of *hla* was revealed in exudates accumulated in a guinea pig model of infection, indicating that SaeRS is able to function independently (Goerke et al., 2001, 2005). The disruption of of SaeRS abolished the *S. aureus*-induced apoptosis and death of epithelial cells, indicating the importance of SaeRS in modulation of bacterial toxicity (Liang et al., 2006). On the other hand, the mutation of *sae* eliminates the transcription and expression of *fnbA* and increases expression of CP5 in Newman strain and consequently decreases the internalization of *S. aureus* by endothelial cells (Steinhuber et al., 2003). We revealed that SaeRS also affects the expression of Efb, a bifunctional protein capable of binding to both extracellular fibrinogen (Peacock et al., 2002) and complement factor C3 (Lee et al., 2004), and the expression of SA1000, a hypothetical fibrinogen-binding protein (Liang et al., 2006). Both Efb and SA1804 contribute to the adherence and internalization of *S. aureus* by epithelial cells (Liang et al., 2006). Importantly, the role of the SaeRS as a virulence regulator has been demonstrated in several animal models of infection (Benton et al., 2004; Goerke et al., 2005; Liang et al., 2006; Montgomery et al., 2010).

It has been demonstrated that SaeRS activates the expression of virulence factors through directly binding to the promoter regions of some virulence gene, such as *hla, emp,* and *map/eap* by phosphorylated response regulator SaeR (Sun et al., 2010). Our previous transcriptomics study indicated that inactivation of SaeRS dramatically increases the transcription of a novel transcriptional regulator (SA1804). This led us to hypothesize that SA1804 is involved in the pathogenesis of *S. aureus*. In this study, we created *sa1804, saeRS, and sa1804/saeRS* double deletion mutants in a USA300 community-acquired MRSA strain, 923, and determined their impact on the pathogenicity.

## Materials and Methods

### Bacterial Strains, Media, and Growth Conditions

*S. aureus* strain RN4220 was utilized as the primary recipient for allelic exchange constructs, together with a virulent clinical isolate WCUH29 (NCIMB40771) and USA300 MRSA isolate 923 (Montgomery et al., 2010), as secondary recipients for electroporation. *Escherichia coli* DH10B (Invitrogen) served as the host for all *in vitro* recombinant DNA. Bacteria were grown in Tryptic Soy broth (TSB; Difco) and on TSA agar at 37°C with appropriate antibiotics. Bacterial cell cultures were incubated at 37°C with shaking at 200 rpm.

### Construction of the *saeRS* and/or *sa1804* In-Frame Deletion Mutants, the *saeRS* and *sa1804* Complementary Strains, and *sa1804* Promoter-*lux* Reporter Fusion

In-frame deletion of *saeRS and/or sa1804* was carried out following the pKOR1 allelic exchange protocol as described (Bae and Schneewind, 2006). The deletion in *saeRS*, *sa1804,* or* saeRS/sa1804* was confirmed diagnostic PCR and DNA sequencing flanking regions of *sa1804*.

In order to examine whether the expression of *saeRS* or* sa1804* in *trans* complements the effect of the mutation of endogenous correspondence gene, we constructed complementary plasmids, including pYH4/*saeRS* and pYH4/*sa1804* by cloning the *saeRS* or* sa1804* coding region (which was obtained by PCR using primers listed in **Table 1**) into the *Asc*I and *Pme*I sites of pYH4 (Ji et al., 2004). The resulting plasmid was electroporated into the *saeRS*ko and *sa1804*ko, respectively, resulting in SaeRScom, and SA1804com strains. To confirm the impact of SaeRS on the transcription of *sa1804*, we created a *sa1804* promoter-*lux* promoter system as described (Yan et al., 2011). The upstream sa1804 promoter region was PCR amplified using the primers listed in **Table 1**, digested with *Eco*RI and *Xma*I (NEB), and cloned into same enzyme sites of pCY1006. The reformed P*sa1804*-*lux* reporter fusion was confirmed by diagnostic PCR and electroporated into the *saeRS*ko and parental control. The P*sa1804*-*lux* reporter fusion strains were grown in TSB at 37°C with shaking overnight. Bioluminescence intensity and optical density of the cultures were measured at different times of the experiment in duplicate. The relative light units (RLU) were calculated by dividing the average bioluminescence reading by the average OD600_nm_ reading (^lum^/_OD600nm_) at each time point. The experiment was repeated three times with separate colonies of each strain.

**Table 1:** Primers used in qPCR.

Primer	Sequence (5′-3′)
Sal804proEcoRfor	ATGAATTCTGGAAGTGATGTCGTTATCG
Sal804proXmarev	TACCCGGGCATGTTGTCACCGCCTTTC
Sal804forPmeI	AGCTTTGTTTAAACTATGAAAACATTAAAAGAGTTGAGG
Sal804RevAscI	TTGGCGCGCCTTAAGATGTTTGTTTTTCTTTAAATGC
Sa1804Ndefor	TGAAAAAACATATGAAAACATTAAAAGAGT TGA G
Sa1804Bamrev	TTGGATCCTTAAGATGTTTGTTTTTCTTTAAA
ProfnbBfor	AAGAATTCGCGCTTTATGTCTGATGATTG
ProfnbBrev	AACCCGGGCTCCCTTAAATGCAAAATTC
SA1000for	GTATCAACGTTTGCCGGTGAATCTC
SA1000rev	CAGCTCTTTGTGCTTTACGGTGTGTT
Efbfor	GTACAATGATGGTACTTTTAAATATCAATCTAGAC
Efbrev	GTTCTTTTTTAATAGTTGCATCAGTTTTCGCT
Hlafor	CAACTGATAAAAAAGTAGGCTGGAAAGTGAT
Hlarev	CTGGTGAAAACCCTGAAGATAATAGAG
FnbB for	GCAGTGAGCGACCATACAACAGTT
FnbBrev	CAATCACGCCATAATTACCGTGACCA
gyrBfor	GGTGGCGACTTTGATCTAGC
gyrBrev	TTATACAACGGTGGCTGTGC

### RNA Purification and Real-Time RT-PCR Analysis

Overnight cultures of *S. aureus* were inoculated in 5% in TSB medium and grown to the mid-exponential (3 h) phase of growth. Cells were harvested by centrifugation; RNA was isolated using the RNAPrep Kit (Promega, MI) as described (Lei et al., 2014). Contaminating DNA was removed with a DNA-*free* Kit (Ambion). The first strand cDNA was synthesized using reverse transcriptase with the SuperScript III Platinum Two-Step qRT-PCR Kit (Life Science Technology). For each RNA sample, duplicate reactions of reverse transcription were performed, as well as a control without reverse transcriptase, in order to determine the levels of DNA contamination. PCR reactions were set up in triplicate by using the SYBR Green PCR Master Mix (Bio-Rad). Real-time sequence-specific detection and relative quantization were performed with the Stratagene Mx3000P Real Time PCR System. Gene-specific primers were designed to yield ∼100 bp of specific products (**Table 1**). Relative quantification of the product will be calculated using the Comparative C_T_ method, as described for the Stratagene Mx3000P system. The housekeeping gene *gyrB* was used as an endogenous control. All samples were analyzed in triplicate and normalized against *gyrB* gene expression. The experiments were at least repeated twice and analyzed for correlation to the microarray results.

### Cell Culture and Epithelial Cell Invasion Assay

A549 human lung epithelial cells (ATCC CCL 185) were cultured in RPMI 1640 medium supplemented with 10% fetal bovine serum (FBS; Invitrogen). Cultures of A549 cells were maintained in a medium containing penicillin (5 μg/ml) and streptomycin (100 μg/ml; Sigma). Bacterial invasion assyas were performed in RPMI 1640 medium with supplement of 10% FBS (RPMI-FBS) as described (Yang and Ji, 2014). Briefly, one day prior to infection, approximately 2 × 10^5^ cells were seeded in each well of 24-well plates, and incubated overnight at 37°C in a CO_2_ incubator. Monolayers of A549 cells (2 × 10^5^ cells/well) were infected by adding 0.5 ml RPMI containing approximately 5 × 10^5^ cfu of bacteria followed a centrifugation at 100 *g* for 5 min, and incubated for 2 h at 37°C in 5% CO_2_. The bacteria outside of the monolayer cells were killed by adding1 ml of RPMI-10% FCS containing 100 μg/ml gentamicin and 5 μg/ml lysostaphin to invasion wells. We used 0.025% TritonX-100 for better cell lysis. The numbers of bacterial cfu released from the lysed epithelial cells were determined by plating of diluted lysates on TSA-agar plates. Each experiment was repeated three times and statistically analyzed by TTEST, using Microsoft Excel software. *P*-values of < 0.05 were considered significant.

### Cytotoxicity Assays

All cells were grown in 96-well plates to 70% confluence. Inhibitors were diluted serially in complete medium and applied to the cells. The supernatants were collected from the overnight cultures, sterilized by filtration, and applied to the epithelial cells, and the treated cells were incubated at 37°C with 5% CO_2_ overnight for 18 h. At the end of the experiment, cell viability was determined using the CellTiter 96^®^ Aqueous Non-Radioactive Cell Proliferation Assay (Promega) as manufacturer’s instructions. Each experiment was repeated three times and statistically analyzed by TTEST, using Microsoft Excel software. *P*-values of < 0.05 were considered significant.

### Cloning, Expression, and Purification of the SA1804-HIS Tagged Fusion Protein in *Escherichia coli*

The *sa1804 gene* was obtained by PCR amplification using the premiers listed in **Table 1**, cloned into pET28a, and resulted in pSA1804-28a. Plasmid pSA1804-28a were introduced into a BL21(DE3) strain. The resulting strains were grown in LB medium at room temperature; the expression of SA1804 was induced when the culture media reached OD600nm equal to 0.6 by addition of 1 mM IPTG (isopropyl-*b*-d-thiogalactoside) and incubation pursued for 4 h. The SA1804-his tagged protein was purified using Ni-NTA agarose column (Novagen) as described (Yan et al., 2011).

### Electrophoretic Mobility Shift Assay

To determining whether SA1804 directly regulates *hla, sa1000, and/or efb* transcription, a DIG-labeled probe of *hla* promoter region was utilized for as described (Liang et al., 2011). The dual 5’-biotin labeled promoter fragments of *sa1000 and efb* were obtained by high-fidelity PCR and gel-purified using a NucleoSpin Gel Clean-up kit (Macherey–Nagel). The purified biotin-labeled probes were utilized for the electrophoretic mobility shift assay (EMSA) using the LightShift Chemiluminescent EMSA Kit (Thermo Scientific). All samples contained 1X LightShift Binding Buffer, 50 ng/μl Poly (dI∙dC), and 2.5% glycerol. The labeled probe, SA1804-His, non-labeled specific probe, BSA, and non-specific non-labeled probe were all added to concentrations as outlined in the figure and ultrapure water was added so that all reaction volumes totaled 20 μl. The reactions were incubated at room temperature for 20 min followed by addition of 5 μl of 5X loading buffer to each reaction. 20 μl of each reaction were loaded into the wells of a pre-run 8% TBE native polyacrylamide gel and electrophoresed at 75 V for three hours at 4°C. The samples were transferred to nylon membrane and processed as outlined in the manufacturer’s protocol. BioMax Light Film (Kodak) was used to detect the chemiluminescent reaction.

## Results

### Transcriptional Repression of *sa1804* By SaeRS

**FIGURE 1:**
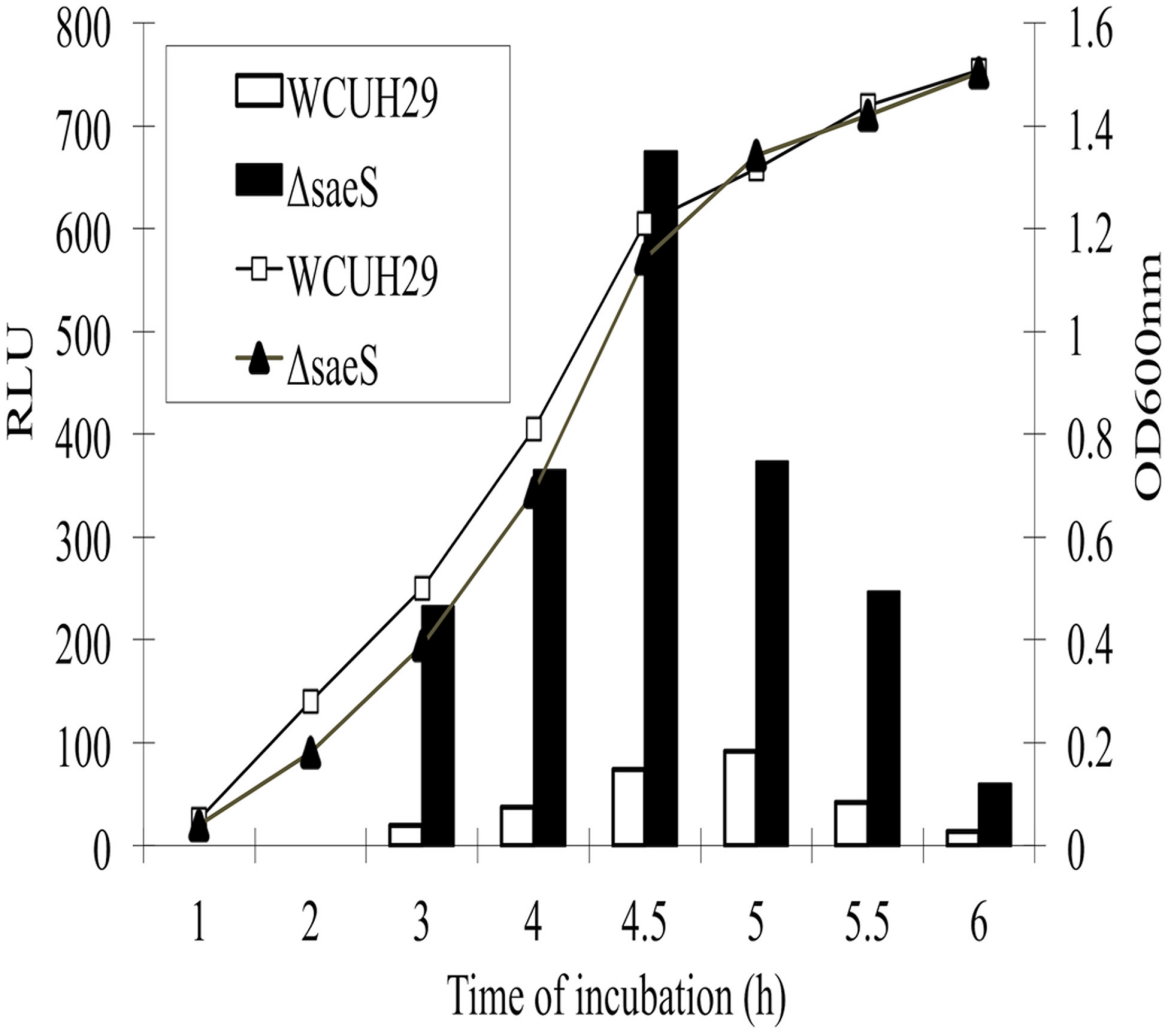
**Effect of the deletion of *saeS* on the* sa1804* promoter transcription activity.** Both bioluminescence signals and cell growth were monitored at different phases of growth at 37°C by measuring the light intensity with a Chiron luminometer and optical density at 600 nm (OD_600_) with a SpectraMax plus Spectrophotometer. Relative light unit (RLU) was calculated with the bioluminescence intensity divided by the optical density in the same time of culture. The experiments were repeated at least three times. Solid column represents the *saeS* deletion mutant and open column represents the parental wild type strain.

Our previous microarray analysis showed that the mutation of *saeS* significantly induced the transcription of a putative regulator gene (*sa1804*), suggesting that SaeR might be a repressor of SA1804 (Liang et al., 2006). To further confirm whether SaeRS system negatively mediates the transcription of *sa1804*, we conducted a P*sa1804*-*lux* reporter experiment. We detected the *sa1804* transcription levels by kinetically measuring the intensity of bioluminescence signal during growth over time. The transcription level of *sa1804* strikingly increased from the mid-log phase of growth and peaked at the early stationary phase of growth in the *saeRS* null mutant compared to the control strain (**Figure 1**). This suggests that SaeRS system transcriptionally modulates the expression of SA1804.

### The Deletion Mutation of *sa1804* Enhances the Capacity of *S. aureus* to Invade Into Epithelial Cells in a SaeRS-Dependent Manner

**FIGURE 2:**
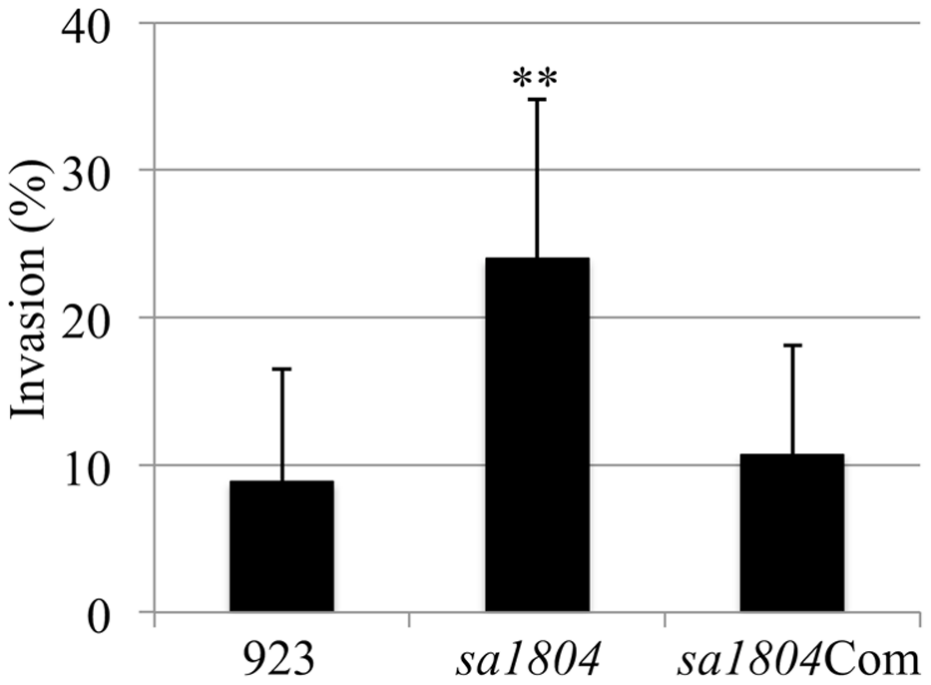
**Effect of the deletion of *sa1804* on the bacterial invasion of the human lung epithelial cell line (A549).** Intracellular invasion was calculated as described in Section “Materials and Methods." Data are the means ± SE of the means from at least three repeated experiments. 923: wild type CA-MRSA control; *sa1804*: *sa1804* deletion mutant; *sa1804*Com: *sa1804* complementary strain. The symbol **represents *P* < 0.01 between wild type control and sa1804 knockout mutant.

Our previous studies demonstrated that the SaeRS is a major regulator of bacterial adhesion and invasion and SaeRS represses *sa1804* transcription. This led us to question the impact of SA1804 on the capacity of bacteria to adhere to and invade into the host cells. To address this question, first we constructed a *sa1804* null mutant using a USA 300 human clinical *S. aureus* isolate 923. Then, we examined the impact of the null mutation of SA1804 on invasion using a human lung epithelial cell line (A549). The results showed that the mutation of *sa1804* significantly elevated the capacity of bacteria to invade into the epithelial cells (**Figure 2**). To confirm the role of Sa1804, we constructed sa1804 complementary strain, and determined whether the expression of Sa1804 *in trans* complements the effect of depletion of SA1804. The results showed that the complementation of Sa1804 alleviated the bacterial ability to invade epithelial cells (**Figure 2**).

**FIGURE 3:**
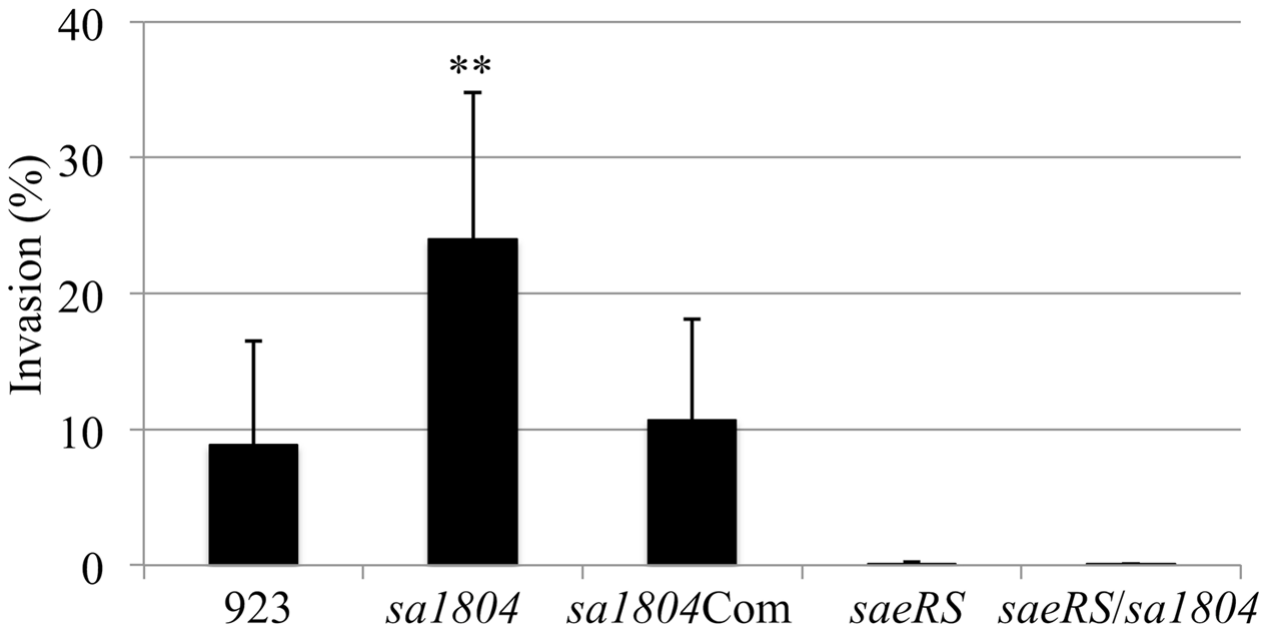
**Effect of the deletion of *saeRS*, *sa1804, or saeRS*/*sa1804* on the bacterial invasion of the human lung epithelial cell line (A549).** Intracellular invasion was calculated as described in Section “Materials and Methods." Data are the means ± SE of the means from at least three repeated experiments. 923: wild type CA-MRSA control; *sa1804*: *sa1804* deletion mutant; *sa1804*Com: *sa1804* complementary strain; saeRS: saeRS deletion mutant; *saeRS*/*sa1804*: *saeRS* and *sa1804* double deletion mutant. The symbol **represents *P* < 0.01 between wild type control and sa1804 knockout mutant.

To elucidate whether SA1804 functions through the regulation of SaeRS, we generated *saeRS and saeRS/* *sa1804* double knockout mutants and examined their impact on bacterial invasion. Consistent with our previous findings the deletion of *saeRS* loci totally eliminated the bacterial capacity of invading of epithelial cells (**Figure 3**). The deletion mutation of *saeRS* eliminated the effect of SA1804 on the invasion ability of in the *sa1804* null mutant (**Figure 3**). The above data indicate that the involvement of SA1804 in regulation of bacterial invasion is dependent on SaeRS.

### The Deletion Mutation of *sa1804* Increases the Cytotoxicity of *S. aureus* to Epithelial Cells in a SaeRS-Dependent Manner

**FIGURE 4:**
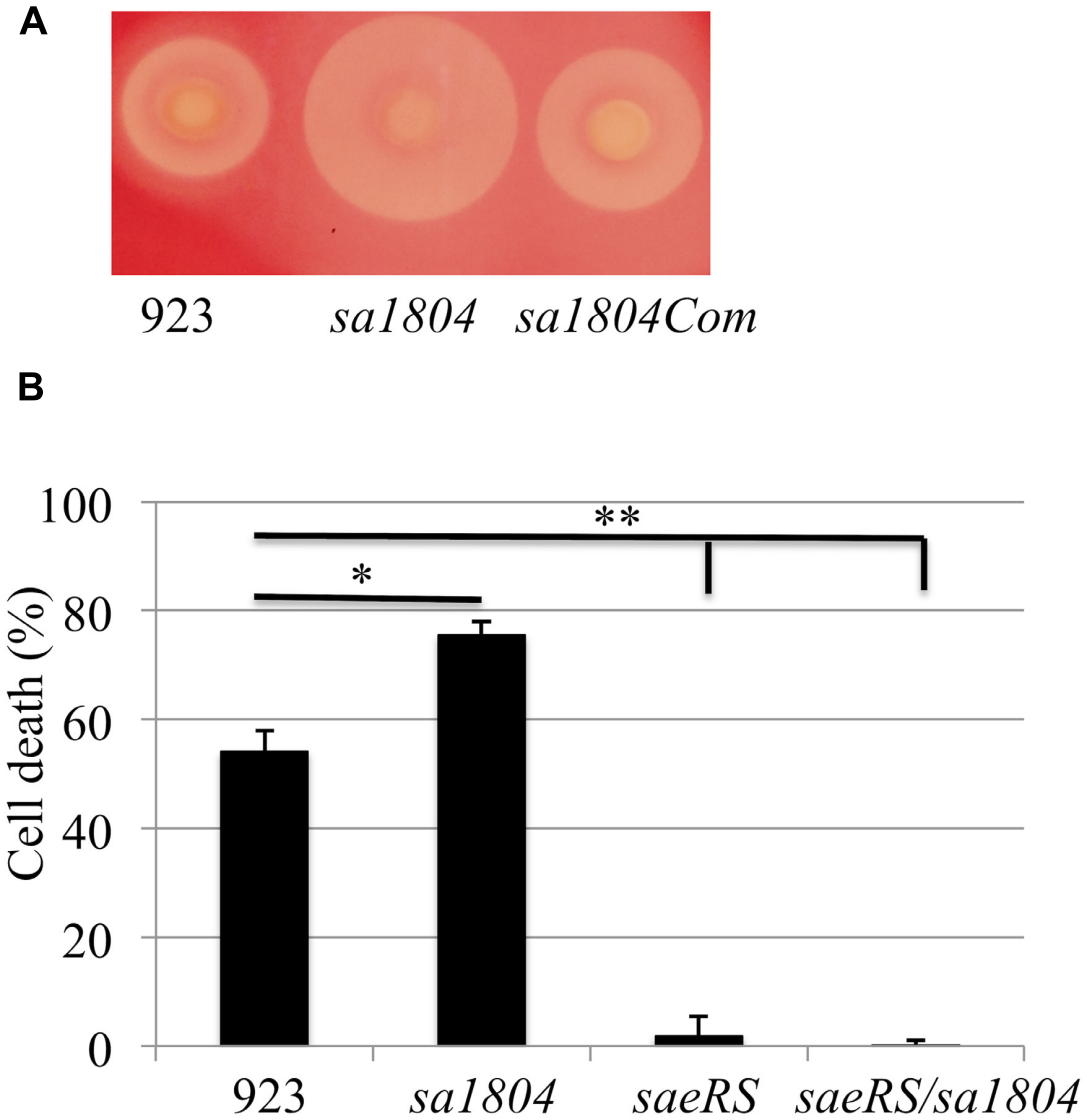
**Effect of SA1804 on bacterial hemolysis and on the bacterial cytotoxicity. (A)** Hemolytic analysis on Sheep Blood Agar. **(B)** Effect of supernatants of overnight cultures on cell death. The supernatants were collected from overnight cultures and sterilized by filtration. Monolayers of A549 cells (2 × 10^5^ cells/well) were exposed to the supernatant of the cultures. Cell viability was measured at 18 h after treatment and is expressed as an average of at least three experiments ± SD. 923: wild type CA-MRSA control; *sa1804*: *sa1804* deletion mutant; *sa1804*Com: *sa1804* complementary strain; *saeRS*: *saeRS* deletion mutant; *saeRS*/*sa1804*: *saeRS* and *sa1804* double deletion mutant. The symbol *represents *P* < 0.05 between wild type control and *sa1804* knockout mutant; the symbol **represents *P* < 0.01 between wild type control and *saeRS* knockout or *saeRS*/*sa1804* double knockout mutant.

To further determine the role of SA1804 in pathogenicity of *S. aureus*, we performed hemolysis and cytotoxicity assays by measuring LDH as described (Liang et al., 2006). The deletion of *sa1804* increased the hemolysis on a sheep blood agar plate, whereas the expression of SA1804 *in trans* complemented the hemolytic activity of *S. aureus* 923 isolate (**Figure 4A**). We found that the supernatant of the wild type strain, 923, caused 54% of A549 cells died 16 h after exposure, whereas 75% of the cells died after exposing to the supernatant from the *sa1804* knockout mutant culture (**Figure 4B**). Deletion of *saeRS* abolished the toxicity of the supernatant of the culture, whereas knockout of *saeRS* had no impact of the deleted *sa1804* on cytotoxicity. This further indicates that the regulator SA1804 plays a role in the regulation of virulence factors in a *saeRS* dependent manner.

### The Putative Transcriptional Regulator (SA1804) Negatively Regulates the Expression of *efb*, sa1000 and *hla* in *S. aureus*

To elucidate potential molecular mechanism behind the impact of SA1804 on bacterial invasion and cytotoxicity, we examined the impact of SA1804 on transcriptions of several potential target genes using qPCR approach. Total RNA was purified from the mid-log phase of cultures of the *sa1804* null mutant, *saeRS* null mutant, *saeRS*/*sa1804* double knockout mutant, and parental control. The transcription levels of selected genes were detected by qPCR. The housekeeping gene *gyrB* was used as an internal control. No difference of *gyrB* gene transcription level was revealed between the *sa1804 and/or saeRS* null mutant and it parental control. However, the transcriptional levels of* hla*, *efb,* and *sa1000* increased approximately threefold in the *sa1804* null mutant than that parental control (**Table 2**). In contrast, the deletion of *saeRS* remarkably decreased these genes transcription. Taken together, the above data demonstrated that the putative regulator SA1804 transcriptionally represses the expression of *hla, efb, and sa1000* in *S. aureus* 923 isolate.

**Table 2:** qPCR analysis of expression of genes regulated by SaeRS.

N315 gene	Description	Fold change *^a^* in *sa1804*ko	Fold change *^a^* in *saeRS*ko	Fold change in s*a1804*/*saeRS*ko
*hla*	Alpha-hemolysin	3.0	-56.49	-40.93
*sal 000*	Hypothetical fibrinogen-binding protein	2.9	-20.38	-20.97
*efb*	Extracellular fibrinogen-binding protein	3.86	-12.91	-11.0
*fnbB*	Fibronectin-binding protein B	-0.96	-1.7	-1.33


*^a^*Normalized values in the knockout mutant over values in the wild type 923 strain. Negative number denotes down-regulated in mutant strain.

### The Putative Regulator Sa1804 Binds the *hla* Promoter Region *in vitro*

**FIGURE 5:**
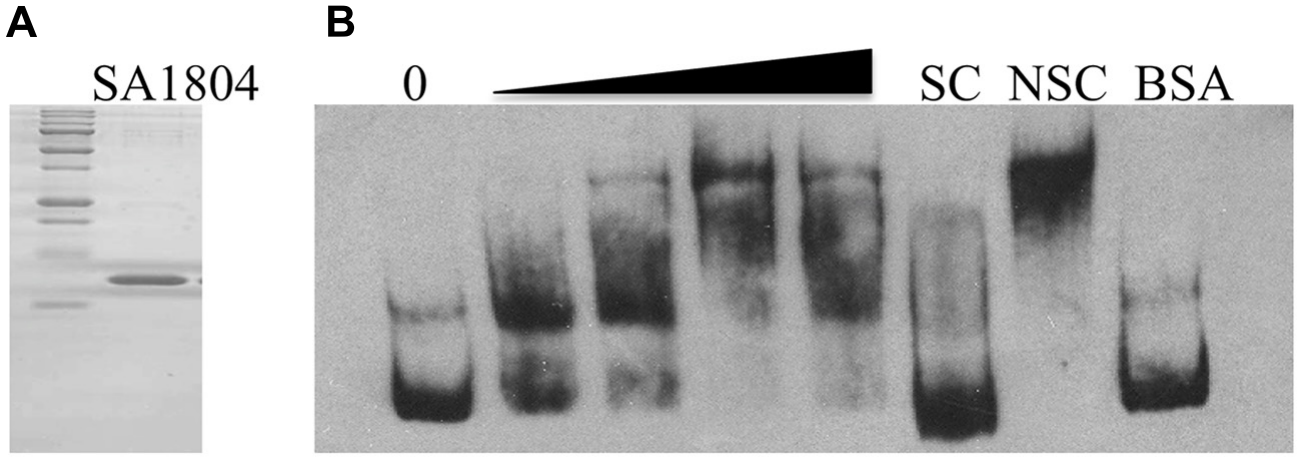
**(A) SDS-PAGE analysis of the purity of purified recombinant SA1804; **(B)** Electrophoretic mobility shift analysis of the hla promoter regulated by SA1804.** The promoter region of the *hla* gene was obtained by PCR, purified, and labeled with Digoxigenin. The mobility of the labeled promoter fragment without addition of SA1804 is shown in the first lane. Different amounts of SA1804 protein (25, 100, 250, 500 ng) were incubated with each labeled *hla* promoter probe in a 15 μl reaction volume. Specific competitor (SC) control: incubation in the presence of 100-fold excess unlabeled SC. Non-specific competitor (NSC) control: incubation in the presence of 100-fold excess unlabeled non-specific internal gene probe. BSA (1 μg) was used as non-specific binding control.

To elucidate the potential mechanism of regulation, we examined whether SA1804 directly or indirectly regulates the transcription of the *hla, efb, and sa1000* genes using gel-shift assays. We cloned, expressed, and purified SA1804-his tag protein as described (**Figure 5A**). BSA protein was used as a control. The promoter regions of *hla*, *efb,* and *sa1000* genes were obtained by PCR, labeled, and purified using PCR cleanup kits. Unlabeled probe was utilized as specific competitor (SC) accordingly. Apparent *hla* probe-SA1804 complex formed in a dose-dependent manner (**Figure 5B**). However, no *hla* probe-SA1804 complex was observed for the *efb* and *sa1000* promoters (data not shown). These suggest that SA1804 may directly mediate *hla* transcription by binding to *hla* promoter region, but indirectly regulate *efb* and *sa1000* transcription.

## Discussion

In this study, we are the first demonstrating that SA1804 is a repressor of several virulence factors, including *hla, efb,* and* sa1000* in a CA-MRSA USA300 isolate 923. Moreover, our data suggest that SA1804 indirectly mediates the transcription of *efb* and* sa1000*, whereas directly controls the transcription of *hla* through binding to the promoter region of *hla*. Furthermore, our data clearly indicate the role of SA1804 in manipulation of the pathogenicity of *S. aureus*, as the null mutation of *sa1804* enhanced the capacity of bacteria internalizing into the epithelial cells and the cytotoxicity. In addition, we further confirmed that SaeRS negatively regulates the transcription of the novel transcriptional regulator SA1804. Our data suggest that the regulatory function of SA1804 is dependent on the activation of SaeRS, as the disruption of SaeRS eliminated the influence of SA1804 on the transcription of selected virulence factors, including *hla*, *efb*, and *sa1000,* as well as the bacterial invasion ability and cytotoxicity.

Our *sa1804* promoter-*lux* reporter fusion experiment showed that the inactivation of SaeRS remarkably elevated the transcription of *sa1804*, which is consistent with our microarray analysis result (Liang et al., 2006). Together with previous findings in different *S. aureus* isolates (Giraudo et al., 1999; Liang et al., 2006; Rogasch et al., 2006), our qPCR analysis further confirmed the effect of SaeRS on positive regulation of key virulence factors, including *hla*, *efb*, *fnbB,* and *sa1000* in CA-MRSA USA300 isolate 923. Moreover, our invasion and cytotoxicity assays demonstrated that SaeRS plays a key role in the pathogenesis of *S. aureus*, which is highly supportive for the importance of SaeRS in pathogenicity in animal models of infection (Liang et al., 2006; Montgomery et al., 2010). Previous reports indicate that SaeRS tightly controls the expression of *fnb* (Giraudo et al., 1999) since no mRNA of *fnbA* and *fnbB* was detectable in the *saeS* mutant strain (Steinhuber et al., 2003). In this study, we found that the deletion mutation of *saeRS* had a weak effect on the transcription of *fnbB*. This discrepancy is likely due to the difference of genetic background among different *S. aureus* isolates.

It was well established that SaeRS is a major regulator of *hla* expression in *S. aureus* (Liang et al., 2006; Montgomery et al., 2010); SaeR can directly regulate the *hla* transcription by binding to its promoter region (Cho et al., 2012; Olson et al., 2013). Although our gel-shift result indicated that the novel transcriptional regulator, SA1804, is able to bind to the *hla* promoter region, our data clearly demonstrated that SaeRS plays a predominant role in the regulation of the *hla* transcription in *S. aureus*.
